# Different Design Feature Combinations of Flatfoot Orthosis on Plantar Fascia Strain and Plantar Pressure: A Muscle-Driven Finite Element Analysis With Taguchi Method

**DOI:** 10.3389/fbioe.2022.853085

**Published:** 2022-03-10

**Authors:** Yinghu Peng, Yan Wang, Duo Wai-Chi Wong, Tony Lin-Wei Chen, Shane Fei Chen, Guoxin Zhang, Qitao Tan, Ming Zhang

**Affiliations:** ^1^ Department of Biomedical Engineering, Faculty of Engineering, Hong Kong Polytechnic University, Kowloon, Hong Kong SAR, China; ^2^ Hong Kong Polytechnic University Shenzhen Research Institute, Shenzhen, China; ^3^ CAS Key Laboratory of Human-Machine Intelligence-Synergy Systems, Shenzhen Institutes of Advanced Technology Chinese Academy of Sciences, Shenzhen, China

**Keywords:** flatfoot, foot orthosis, finite element model, plantar fascia, Taguchi approach

## Abstract

Customized foot orthosis is commonly used to modify foot posture and relieve foot pain for adult acquired flexible flatfoot. However, systematic investigation of the influence of foot orthotic design parameter combination on the internal foot mechanics remains scarce. This study aimed to investigate the biomechanical effects of different combinations of foot orthoses design features through a muscle-driven flatfoot finite element model. A flatfoot-orthosis finite element model was constructed by considering the three-dimensional geometry of plantar fascia. The plantar fascia model accounted for the interaction with the bulk soft tissue. The Taguchi approach was adopted to analyze the significance of four design factors combination (arch support height, medial posting inclination, heel cup height, and material stiffness). Predicted plantar pressure and plantar fascia strains in different design combinations at the midstance instant were reported. The results indicated that the foot orthosis with higher arch support (45.7%) and medial inclination angle (25.5%) effectively reduced peak plantar pressure. For the proximal plantar fascia strain, arch support (41.8%) and material stiffness (37%) were strong influencing factors. Specifically, higher arch support and softer material decreased the peak plantar fascia strain. The plantar pressure and plantar fascia loading were sensitive to the arch support feature. The proposed statistics-based finite element flatfoot model could assist the insole optimization and evaluation for individuals with flatfoot.

## Introduction

Individuals with flatfoot may demonstrate abnormal foot kinematics, such as excessive rearfoot eversion, collapsed foot arch, valgus forefoot, altered muscle activations, and potential symptoms (e.g., knee pain and foot pain) (Richie Jr, 2007; Cifuentes-De la Portilla et al., 2019; Flores et al., 2019; Zhang and Lu, 2020). In the early stages of the disease, conservative treatments are often prescribed to alleviate foot pain and modify the foot posture (Lee et al., 2005; Arachchige et al., 2019; Peng et al., 2020; Cen et al., 2021). Foot orthoses, one of the most common conservative treatments, have been widely used to provide support, distribute the foot pressure, correct the flexible misalignment, and constrain the painful joints (Cobb et al., 2011; Telfer et al., 2013; Banwell et al., 2014; Kosonen et al., 2017).

Studies have investigated the effects of foot orthoses on the kinematic and kinetics of flexible flatfoot (Chen Y.-C. et al., 2010; Hurd et al., 2010; Murley et al., 2010; Cobb et al., 2011; Kosonen et al., 2017). Foot orthoses with arch support are common considerations for redistributing plantar foot pressure and relieving foot pain (Cheung and Zhang, 2005). However, the prescribed foot orthoses with enhanced arch support alone cannot reduce the excessive rearfoot eversion. Medial forefoot and rearfoot postings were considered in addition to arch support for more aggressive flatfoot posture correction (Desmyttere et al., 2018). Although previous studies have proposed and tested various foot orthoses for flatfoot, there is a lack of consensus on the foot orthoses design and their configurations on foot kinematics and kinetics (Cobb et al., 2011; Telfer et al., 2013; Banwell et al., 2014; Kosonen et al., 2017). Compared to prefabricated foot orthosis, customized foot orthoses can provide better functional outcomes in hindfoot correction and pain relief due to their consideration in individual variance (Cheung et al., 2011). However, design feature combinations for the customized foot orthosis seriously depended on the experience of a pedorthist, lacking quantitative theoretical support.

Kinematics and kinetics studies were conducted to investigate the effects of orthosis and provide knowledge-based information for flatfoot intervention (Kogler et al., 1995; 1996; Murley et al., 2014). However, although gait analysis could reveal the kinematics changes induced by foot orthosis, the internal foot soft tissue stress loading distribution was hardly obtained (Murley et al., 2014). Meanwhile, cadaver studies and dynamics Magnetic Resonance Imaging (MRI) or biplane fluoroscopy methods had difficulty in analyzing the effects of material sensitivities and experimental setting (Kogler et al., 1995). Compared to these techniques, the finite element (FE) analysis approach offers an alternate approach for internal soft tissue stress investigation, which may be useful in gaining new insights into the mechanisms related to pathomechanics in musculoskeletal systems (Wang et al., 2016).

Foot-ankle complex FE models have been adopted to investigate the effects of orthotic insoles on foot plantar pressure (Cheung and Zhang, 2008; Hsu et al., 2008; Chen W.-M. et al., 2015). One study has predicted an optimal insole to lower the plantar fascia stress and peak plantar pressure (Hsu et al., 2008). Another study also investigated the biomechanical effects of the material hardness and support height and reported that higher arch support increased the long plantar ligament stresses (Su et al., 2017). However, these studies only considered the balanced standing condition, and the effects of foot orthoses on the internal force of flatfoot during walking have not been investigated. Meanwhile, previous studies usually adopted a simplified plantar fascia model and could not demonstrate plantar fascia loading (Cheng et al., 2008; Hsu et al., 2008; Yu et al., 2016). Furthermore, although some studies used detailed three-dimensional plantar fascia (Chen Y.-N. et al., 2015; Akrami et al., 2018), these models ignored the interaction between bulk soft tissue and plantar fascia, which might underestimate the plantar fascia loading, especially for orthosis intervention (Peng et al., 2021b). Meanwhile, previous studies only considered limited design parameters in flatfoot intervention (Su et al., 2017), and systematic investigation of the flatfoot loading responses to various orthotic design combinations (e.g., arch support heights, medial posting angles, materials, and heel cup height) remains scarce.

This study aimed at determining how the foot-ankle complex responded to various orthoses design combinations. However, all factor and level combinations need to be analyzed and investigated to determine the impact of the multi-factor, which is time-consuming and costly with the conventional testing approach (Karna and Sahai, 2012). The Taguchi method is a widely used multi-factor and multi-level experimental method based on the orthogonal array (Karna and Sahai, 2012). The results of orthogonal experimental design can achieve a balanced comparison of levels of any factor with less effort (Cheung and Zhang, 2008). Taguchi methods’ analysis has been used to investigate the sensitivity of the design parameters in FE foot models (Cheung and Zhang, 2008; Zhang et al., 2020). Therefore, this study aims to investigate the effects of foot orthosis parameters on the foot pressure and plantar fascia loading distribution at midstance through the Taguchi approach analysis.

## Materials and Methods

**FIGURE 1:**
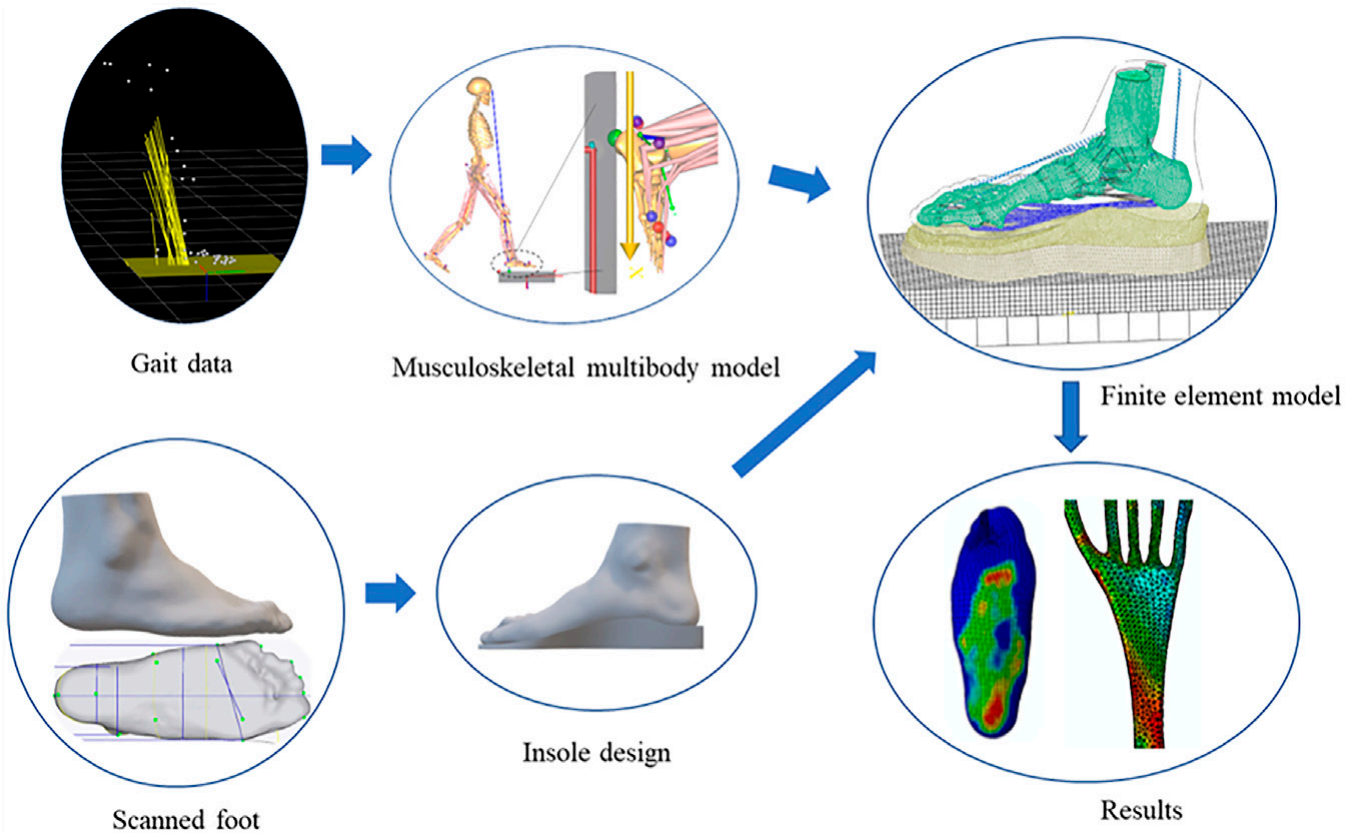
Overview of the foot-ankle complex and insole finite element model. The customized foot orthosis was produced based on the scanned foot surface. Nine design configurations of the foot orthoses were used in the finite element model. Gait data were used as inputs of the musculoskeletal multibody model to calculate the foot muscle forces. The calculated muscles and ground reaction forces were adopted to drive the flatfoot-orthosis finite element model. The foot pressure distribution and plantar fascia strain distribution were predicted based on the muscle-driven foot-ankle complex model.

This study constructed a flatfoot-orthoses FE model with 3D plantar fascia geometry based on foot MRI. The measured ground reaction forces from gait analysis and estimated muscle forces from the musculoskeletal multibody model were used to drive the FE flatfoot model (Peng et al., 2021c). The foot orthoses with four design parameters were adopted in the FE model. Under various foot orthosis design combinations, foot plantar pressure and plantar fascia strain were investigated. More details about the workflow of the customized flatfoot-orthosis modeling can be seen in Figure 1. The statistics-based Taguchi method was used to investigate the sensitivity of four design parameters on the plantar fascia strain and plantar pressure.

### Flatfoot Finite Element Model Construction

The construction of the flatfoot FE model has been reported in our previous studies (Peng et al., 2021a). A young male adult (27 years old, 175 cm height, and 64 kg weight) was recruited for the FE modeling. The participant had flexible flatfoot with an arch index of 0.30 and a navicular drop of 12 mm for the right foot. This study has been approved by the Human Subject Ethics Sub-Committee of The Hong Kong Polytechnic University (Number: HSEARS20190124008). The three-dimensional geometries of the foot-ankle complex were reconstructed using medical image processing software (Mimics 10.1, Materialise Inc., Belgium), including encapsulated bulk tissue, plantar fascia, and twenty bones. The skin layer was defined as a 2 mm thick membrane encapsulating the bulk soft tissue. The inner foot ligaments were modeled as trusses. The contact properties at the joints were assigned with a frictionless contact algorithm with a non-linear contact stiffness to mimic the articular cartilage (Wong et al., 2018). The finite elements of the foot-ankle complex geometries were created by Abaqus 6.14 (Simulia, Dassault Systemes, France). The bones, the encapsulated bulk tissue, orthosis, shoe, and plantar fascia adopted linear tetrahedral elements (C3D4). The hexahedra elements (C3D8), three-node triangular membrane elements (M3D3), and two-node linear three-dimensional elements (T3D2) were assigned to the ground plate, skin, and linear ligaments. The material properties for the foot-ankle complex were determined from current studies (Chen et al., 2003; Lewis, 2003; Chen W.-M. et al., 2010; Peng et al., 2021c). More detailed information about the material properties is shown in Table 1.

**TABLE 1:** Material properties of the components in the finite element model.

	Elastic modulus (MPa)	Poisson ratio	Cross section (mm^2^)
Skin	First-order Ogden hyperelastic model (*μ* = 0.122 MPa, *α* = 18, thickness: 2.0 mm)	—	
Bulk soft tissue	Second-order polynomial strain hyperelastic model (C_10_ = 0.8556, C_01_ = −0.05841, C_20_ = 0.03900, C_11_ = −0.02319, C_02_ = 0.00851, D_1_ = 3.65273)	—	
Bone	10,000	0.34	—
Ligaments	260	0.4	18.4
Three-dimensional Plantar fascia	350	0.45	—
Midsole	5	0.4	—

Parameters for the material property were based on the same references in our previous work (Peng et al., 2021c).

### Load and Boundary Conditions

The internal foot biomechanics were investigated through the muscle-driven flatfoot FE model at the midstance instant during walking. In this model, the proximal cross section surfaces of the fibula, tibia, and bulk soft tissue were fixed. To control the degree of freedom of the ground plate, one rigid plate was tied beneath the ground plate. The ground reaction forces were applied to the rigid plate, including vertical force (338 N), mediolateral force (6 N in the medial direction), and anteroposterior force (4 N in the posterior direction). In addition, the foot muscles forces for the tibialis anterior (38 N), tibialis posterior (200 N), peroneus brevis (1 N), peroneus longus (0 N), Achilles tendon (gastrocnemius and soleus) (774 N), flexor hallucis longus (95 N), and flexor digitorum longus (26 N) were used as inputs to drive the FE model. The musculoskeletal model was adopted to estimate these muscle forces based on the experimental gait data (Peng et al., 2021a). These simulations were conducted with Abaqus 6.14 (Dassault Systèmes, Vélizy-Villacoublay, France) using the standard static solver. The foot plantar pressures and plantar fascia strain were reported.

### Experimental Validation

The muscle-driven flatfoot FE has been validated by comparing the predicted foot pressure with the measurements in our previous studies under barefoot and shod-walking conditions (Peng et al., 2021a; Peng et al., 2021c). The correlation analysis showed that the measurement and prediction were highly associated under barefoot walking (*r* = 0.95, *p* < 0.001) and shod-walking (*r* = 0.8, *p* < 0.001) conditions (Peng et al., 2021a; Peng et al., 2021c).

### Parametric Analysis Through Taguchi Method

**FIGURE 2:**
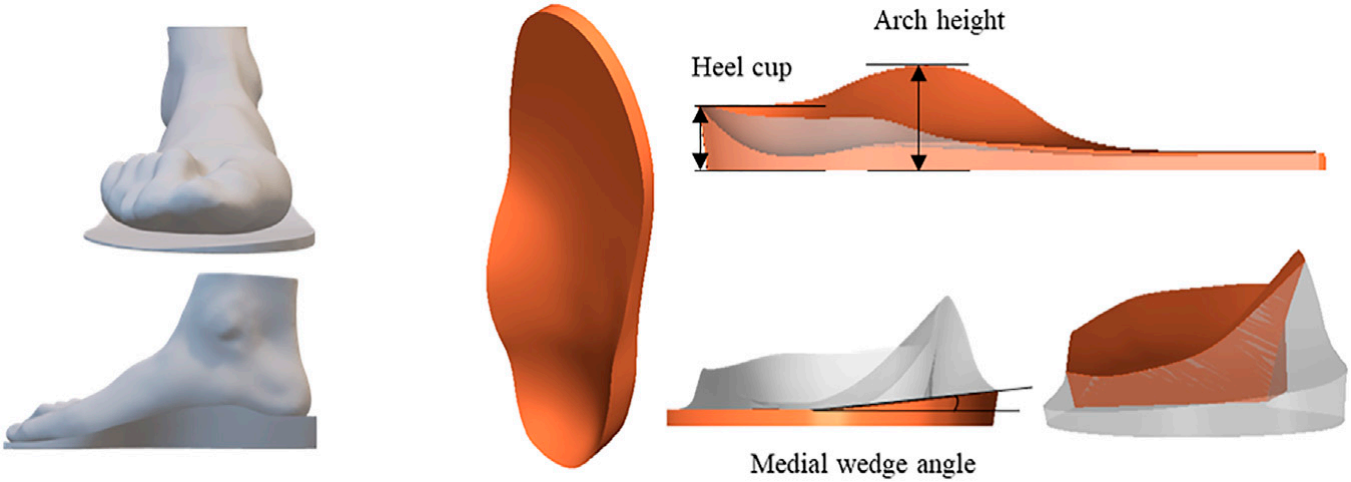
The foot orthosis design parameters, including heel cup, arch support height, and medial wedge angle.

The customized total contact foot orthosis was produced based on the surface scans of the foot under minimal weight (<5% body weight) condition, in which the arch shape was close to the normal configuration. According to the foot shape, the insole profile was designed in the computer-aided design software, isoleCAD (Nmotion Orthotic Lab, Knoxville, TN, United States ). Four design factors, namely, the arch support height (A), the inclination angle of the medial posting (I), heel cup height (H), and material stiffness (M) of insoles, were selected for evaluation. More details of the orthosis configurations are shown in Figure 2. Each factor was assigned with three levels (Table 2).

**TABLE 2:** Foot orthosis design factors and their levels.

Design factor	Level 1	Level 2	Level 3
Arch support height (mm)	42	45	48
Medial posting inclination (°)	0	2	4
Heel cup height (mm)	14	16	18
Materials (MPa)	3	5	7

In this study, a statistics-based Taguchi method was used to reduce the number of analyses. Nine simulations were required. The insole configurations are shown in an orthogonal array L_9_ in Table 3. The mechanical responses, namely, peak plantar fascia strain and peak pressures of the forefoot, midfoot, and hindfoot, were predicted by nine FE analyses. The mean effect of each level of the four design factors on the mechanical responses was computed. For example, the mean response of arch support height at level 1 [R (A_1_)] on peak forefoot pressure is calculated as the mean pressure over trials 1–3. An analysis of variance (ANOVA) was performed, calculating the sum of squares of each design factor to determine the sensitivity of each design parameter (Lee and Zhang, 2005). For example, the sum of squares due to arch support height would be equal to

$${\left[R\left(A_{1}\right)-R_{m}\right]}^{2}+{\left[R\left(A_{2}\right)-R_{m}\right]}^{2}+{\left[R\left(A_{3}\right)-R_{m}\right]}^{2},$$ (1)

where R (A_1_), R (A_2_), and R (A_3_) are mean responses of thickness at levels 1–3, respectively. R_m_ is the overall mean response over nine trials.

**TABLE 3:** L_9_ orthogonal array table (the numbers under design factors indicate the levels assigned to each design factor) and the corresponding FE predicted peak plantar pressures and peak proximal plantar fascia strain for the nine configurations of foot orthosis.

Trial number	Code	Design factor				Plantar pressure (MPa)			Plantar fascia strain (%)
		Arch support	Inclination angle	Heel cup height	Material stiffness	Forefoot	Midfoot	Hindfoot	
		A	I	H	M				
1	A_1_I_1_H_1_M_1_	1	1	1	1	0.183	0.078	0.04	2.16
2	A_1_I_2_H_2_M_2_	1	2	2	2	0.184	0.080	0.078	2.03
3	A_1_I_3_H_3_M_3_	1	3	3	3	0.188	0.096	0.092	2.11
4	A_2_I_1_H_2_M_3_	2	1	2	3	0.187	0.082	0.051	2.19
5	A_2_I_2_H_3_M_1_	2	2	3	1	0.18	0.078	0.047	1.73
6	A_2_I_3_H_1_M_2_	2	3	1	2	0.168	0.109	0.02	1.85
7	A_3_I_1_H_3_M_2_	3	1	3	2	0.18	0.084	0.058	1.48
8	A_3_I_2_H_1_M_3_	3	2	1	3	0.17	0.118	0	2.06
9	A_3_I_3_H_2_M_1_	3	3	2	1	0.164	0.105	0	1.61

## Results

### Plantar Pressures

**FIGURE 3:**
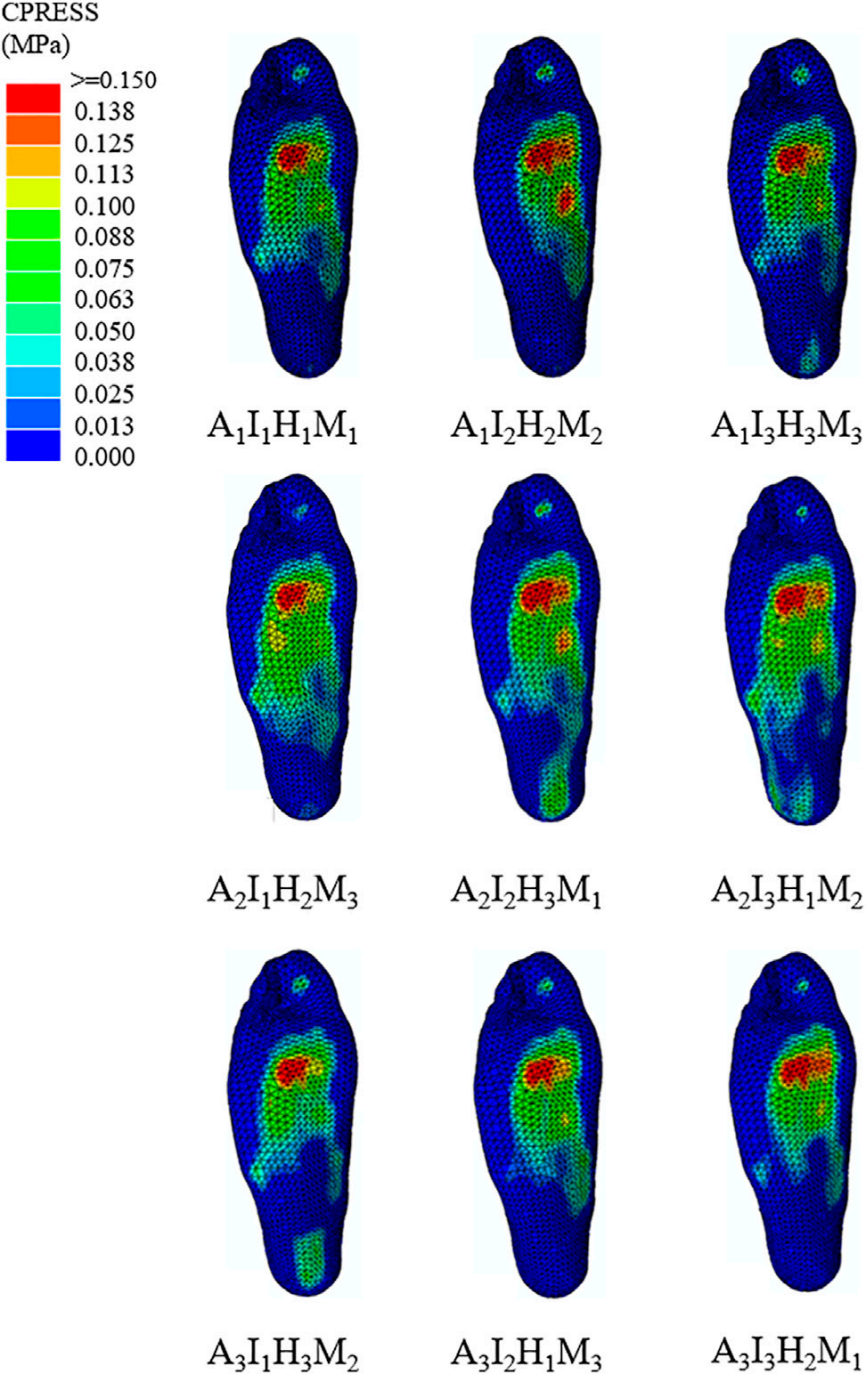
An illustration of the foot pressure distribution among the nine orthoses design configurations.

The FE predicted foot pressure distributions of the nine different orthotic configurations illustrated in Figure 3. The predicted foot plantar pressure was divided into hindfoot, midfoot, and forefoot regions. The peak foot pressures among different orthotic designs occurred in the forefoot region at the midstance instant. The peak foot pressures among three regions for nine orthotic configurations are presented in Table 3. The mean effect of each design factor at each of the three levels can be found in Figure 5. In general, the foot orthoses with higher arch support and medial inclination angle effectively reduced peak plantar pressure. Meanwhile, using a softer material and lower heel cup also reduced the peak foot pressures. The peak plantar pressure of the midfoot increased with higher arch support, medial inclination angle, and material stiffness. However, the high heel cup height decreased the midfoot pressure. In the hindfoot region, foot orthosis with increased medial inclination angle and arch support reduced the peak pressure, and the latter had a larger reducing effect. However, higher heel cup height and material stiffness increased the peak hindfoot pressures. The material stiffness had less effect on the peak hindfoot pressures, especially in the latter two levels.

The degree of importance for each design factor can be further identified by comparing the sum of squares of the predicted plantar pressure shown in Table 4. The geometrical parameters (arch support height, medial posting inclination, and heel cup height) had larger effects on the peak foot pressures than the material stiffness. Specifically, arch support height and medial posting inclination had larger effects on the peak forefoot and midfoot pressures. In contrast, arch support height and heel cup were more important parameters for the peak hindfoot pressures. Among the four design factors, the use of an arch supporting foot orthosis was found to be the most critical design factor for peak foot pressure reduction (45.7%). The medial inclination angle was found to be the second most important factor for peak pressure reduction (25.5%). Then, the rest of the design factors contributed to a much lesser extent in peak pressure reduction with a descending order from heel cup height and insole stiffness. In the midfoot region, the medial posting inclination is the most critical parameter for peak pressure (38.4%). The effects of arch support height and material stiffness are similar (26.7% and 23.1%, respectively). For the hindfoot region, the arch support height had the most effect (47.6%) on the peak hindfoot pressure, followed by heel cup height (37.8%).

**TABLE 4:** Analysis of variance of predicted peak plantar pressure in the forefoot, midfoot, and rearfoot and plantar fascia strain for the four-factor and three-level fractional factorial analysis.

Design factor	Sum of squares for plantar pressure and plantar fascia strain			
	Forefoot	Midfoot	Hindfoot	Plantar fascia
Arch support height	9.5 × 10^−5^ (45.7%)	16.8 × 10^−5^ (26.7%)	125.5 × 10^−5^ (47.6%)	7.4 × 10^−2^ (41.8%)
Medial posting inclination	5.3 × 10^−5^ (25.5%)	24.1 × 10^−5^ (38.4%)	7.6 × 10^−5^ (2.9%)	0.5 × 10^−2^ (2.7%)
Heel cup height	4.1 × 10^−5^ (19.7%)	7.3 × 10^−5^ (11.7%)	99.8 × 10^−5^ (37.8%)	3.3 × 10^−2^ (18.5%)
Materials	1.9 × 10^−5^ (9.1%)	14.5 × 10^−5^ (23.1%)	30.8 × 10^−5^ (11.7%)	6.5 × 10^−2^ (37%)

### Plantar Fascia Strain

**FIGURE 4:**
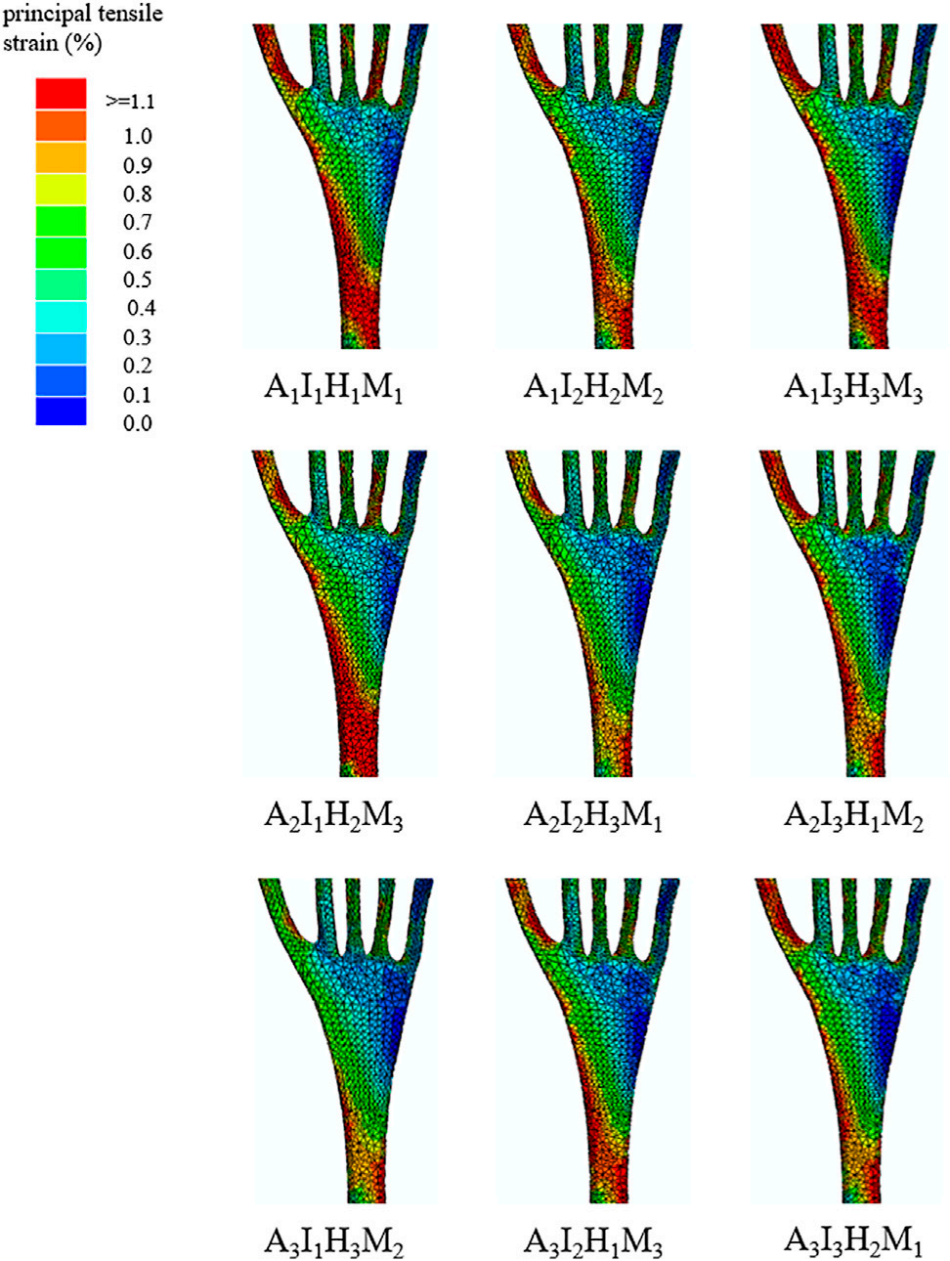
An illustration of the plantar fascia strain distribution among the nine orthoses design configurations.

The predicted principal tensile strain distributions of plantar fascia among the nine orthotic configurations are illustrated in Figure 4. The predicted principal tensile strain distributions of plantar fascia could be divided into the distal, middle, and proximal regions. The peak values in the proximal region among different orthotic designs were reported since plantar fasciitis normally induced pain in the hindfoot region (Cole et al., 2005; Hsu et al., 2008). The peak proximal plantar fascia strain among nine orthotic configurations is presented in Table 3.

**FIGURE 5:**
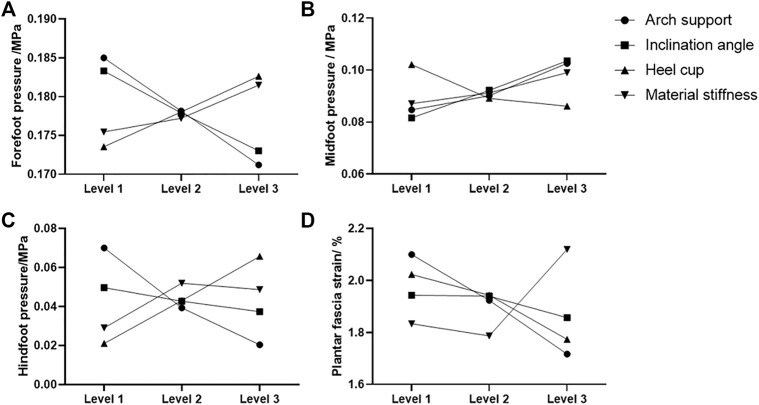
Mean effect of the four design factors at each level on the predicted peak plantar pressure at the **(A)** forefoot, **(B)** midfoot, **(C)** rearfoot regions, and **(D)** predicted proximal plantar fascia strain.

The mean effect of each design factor at each of the three levels can be found in Figure 5. In general, the peak strain of the plantar fascia was decreased with higher arch support, medial inclination angle, and heel cup. The importance degree for each design factor can be further identified by comparing the sum of squares of the predicted plantar fascia strain shown in Table 4. Among the four design factors, arch support (41.8%) and material properties (37%) were significant design factors affecting the peak plantar fascia strain more than other factors.

## Discussion

This study investigated the influence of four orthosis design parameters (arch support height, medial posting inclination, heel cup height, and material stiffness) configurations on foot pressure and plantar fascia loading at midstance. The sensitivity of the foot orthosis design combinations indicated that the foot orthoses with higher arch support and medial inclination angle were more effective in reducing peak plantar pressure. For the proximal plantar fascia, arch support and material properties were significant design factors affecting the peak plantar fascia strain more than other factors. These results could contribute to the customized foot orthosis optimization in clinical practice for flat-arched individuals with symptoms.

The previous study normally indicated that arch support height was the first choice for individuals with flatfoot (Su et al., 2017; Wahmkow et al., 2017). Customized foot orthoses with arch support design could maintain the medial longitudinal arch, elevate the arch height, and distribute foot pressure during stance and walking conditions (Su et al., 2017; Peng et al., 2021c). This study indicated that foot orthosis with higher arch support effectively reduced peak plantar pressure. The peak foot pressure occurred on the forefoot region, one of the most critical areas of clinical significance (Tang et al., 2015). A previous study also indicated that total contact insole reduced the peak forefoot pressures in flat-arched patients, which may partially account for the positive symptom-relieving outcomes after customized foot orthoses intervention (Tang et al., 2015). However, the increased arch support height inevitably increased the pressure of the medial midfoot area, which could cause pain in the medial midfoot region after long-term excessive loading. Foot orthoses with excessive arch support height could also cause excessive stress on the joint cartilage and ligaments of the foot-ankle complex (Su et al., 2017). Therefore, foot orthoses with higher arch support height should be carefully adopted based on the symptoms of the foot.

Although foot orthoses with arch support could resist the collapse of the medial longitudinal arch, it should also be noted that this design alone cannot effectively modify the excessive rearfoot eversion (Desmyttere et al., 2018). Some studies have adopted additional foot orthoses design parameters, such as medial posting, to correct the foot misalignment and distribute the foot pressure (Kosonen et al., 2017; Desmyttere et al., 2018). In this study, the foot orthosis with medial inclination angle was also effective in reducing peak plantar pressure. Compared to foot orthoses with only arch support, this study adopted an additional medial posting design, which was believed to effectively control the rearfoot eversion (Desmyttere et al., 2018). Although medial posting could control the rearfoot eversion, the excessive medial inclination angle should be avoided as the medial posting can have adverse effects. One study has investigated the dose-response effects of medial posting and found that greater medial posting inclination angle increased the external knee adduction moment (Telfer et al., 2013). The increased knee adduction moment could elevate the risk of medial compartment knee osteoarthritis, especially for the elderly (Creaby et al., 2010). In such a case, the clinical symptoms of foot and knee joints should be considered in clinical practice when adopting the medial posting design in the flatfoot intervention.

Different customized orthotic design parameters could be combined to achieve better outcomes, including reduced peak foot pressure and stable foot arch. The material properties can affect foot pressure distribution and medial longitudinal arch stability for customized foot orthoses. Harder insole material can lead to a higher arch height and peak foot pressure (Su et al., 2017). This study also found that stiffer orthoses increased the peak foot pressure. Soft material could be adopted in the specific region to reduce the peak foot pressure in the clinically significant area (Cheng et al., 2021). Meanwhile, the low heel cup can also reduce the peak foot pressure. Therefore, the interaction of the insole shape and materials should be considered in insole optimization for flatfoot patients.

The plantar fascia is one of the most significant passive stabilizers in maintaining the medial longitudinal arch. To identify the plantar fascia loading distribution, this study has adopted the three-dimensional plantar fascia model and considered its interaction with bulk soft tissues, which could withstand the supporting force induced by the ground or insole interface (Peng et al., 2021a; Peng et al., 2021c). The progression of flatfoot deformity could increase the plantar fascia strain and may cause plantar fasciitis (Irving et al., 2006). Previous studies revealed that customized insoles could relieve pain and improve function in runners with running-related overuse injuries (Hirschmüller et al., 2011; Wahmkow et al., 2017). This study indicated that increased arch support could decrease the strain in the proximal region of the plantar fascia. Meanwhile, increased medial posting angle and heel cup height reduced the peak plantar fascia strain in the proximal regions. The influences of arch support height could be more critical than medial posting inclination angle and heel cup height. The reduced plantar fascia strain of the proximal area could account for pain relief in the hindfoot areas (Hsu et al., 2008). The medial posting and heel cup features may be used in conjunction with the arch support design in clinical practice, especially for flat arch individuals with heel pain.

This study has some limitations that should be considered when interpreting the findings and applying them in clinical practice. Firstly, this study adopted the single-subject design for the FE analysis and could ignore the population variability. Because creating a foot-ankle complex involves highly complex boundary and loading conditions, the single-subject models are often used in previous foot-related studies (Wong et al., 2018; Chen et al., 2020; Wong et al., 2020). In this study, we aimed to choose a representative participant of this population to account for the generalizability and endeavored to compromise the external validity issue (Wong et al., 2021). Secondly, the material properties for the specific foot orthoses were the same. Foot orthoses with the same material properties had difficulty achieving reduced foot pressure and stable medial longitudinal arch simultaneously. Future studies could divide the foot orthoses into different regions and adjust material characteristics using different infill rates and patterns with 3D printed techniques. For example, the arch support region could adopt stiffer material, and other regions could adopt softer materials to reduce the peak foot pressure (Cheng et al., 2021). Thirdly, this study has not considered the body weight and foot width when investigating the effects of the arch support height on the foot pressure of the flatfooted participant. Further study should adopt a normalized arch index by simultaneously considering the foot width, foot length, and bodyweight (Lung et al., 2009; Cen et al., 2020). In such a case, the peak pressure could be effectively reduced. Fourthly, this study did not evaluate the foot ligaments stiffness. The foot ligament’s laxity could affect the medial longitudinal arch and internal foot loading (Wong et al., 2020). Further simple clinical tests, such as ultrasound evaluation (Chen et al., 2019), could be performed to obtain the level of generalized ligament laxity or hypermobility of the foot, thus facilitating the foot orthoses design.

## Conclusion

This study investigated the influence of different orthotic design configurations on foot pressure and plantar fascia loading at midstance instant through the muscle-driven FE-orthosis flatfoot model and Taguchi approach. This study adopted a three-dimension plantar fascia geometry, and the interaction between fascia and surrounding soft tissues was considered. The results indicated that the foot orthosis with higher arch support and medial inclination angle was more effective in reducing peak plantar pressure. Meanwhile, medial forefoot posting could be added to modify the forefoot deformity and forefoot pressure. For the proximal plantar fascia, arch support and material properties were more significant design factors for peak plantar fascia strain. Specifically, higher arch support and softer material decreased the peak plantar fascia strain. The statistic-based FE method could provide knowledge-based criteria for designing foot orthosis and fabrication, which can be applied in clinical and commercial settings to treat flatfoot.

## Data Availability

The raw data supporting the conclusion of this article will be made available by the authors without undue reservation.
